# ﻿Notes on the genus *Thibetana* (Lepidoptera, Zygaenidae) with description of a new species from China

**DOI:** 10.3897/zookeys.1218.136369

**Published:** 2024-11-25

**Authors:** Xinxin He, Chao Jiang, Weichun Li

**Affiliations:** 1 College of Agronomy, Jiangxi Agricultural University, Nanchang 330045, China; 2 State Key Laboratory for Quality Ensurance and Sustainable Use of Dao-di Herbs, National Resource Center for Chinese Materia Medica, China Academy of Chinese Medical Sciences, Beijing 100700, China; 3 Jiangxi Provincial Key Laboratory of Conservation Biology, Jiangxi Agricultural University, Nanchang 330045, China

**Keywords:** New species, Procridinae, taxonomy, Xizang, Zygaenidae

## Abstract

The genus *Thibetana* Efetov & Tarmann, 1995 includes six species occurring in southwest China and Indian Sikkim. In the present paper, *Thibetanaweii* Li & He, **sp. nov.**, the seventh species of the genus encountered at the foot of Galongla Snow Mountain, southeast Xizang of China is described. Habitus of the adult and genitalia of the new species are illustrated, and a checklist and a key of all *Thibetana* species are provided.

## ﻿Introduction

The genus *Thibetana* Efetov & Tarmann, 1995 (Zygaenidae, Procridinae) was established to place *Artonasieversi* Alphéraky, 1892 (Efetov and Tarmann 1995). The genus includes six species with an Oriental distribution, occurring in southwest China and Indian Sikkim (Efetov 1997; Efetov and Tarmann 2017; Efetov and Tarmann 2024). Prior to this study, three *Thibetana* species were known from China (Alberti 1954; Efetov 1997; Efetov and Tarmann 2017). The present study aims to add a seventh species to the genus based on specimens collected from Xizang, China.

## ﻿Material and methods

Specimens were hand-collected at night with a 250W mercury-vapour lamp and killed with ammonium hydroxide. The holotype is deposited in the Insect Museum, Jiangxi Agricultural University, Nanchang, China (JXAUM), and the paratype is deposited in the National Zoological Museum of China, Institute of Zoology, Chinese Academy of Sciences, Beijing, China (NZMCAS).

Terminology for morphological structures follows Efetov (1997). Photographs of adults were taken with a Zeiss AxioCam Icc 5 camera attached to a Zeiss Stereo Discovery V12 microscope. Illustrations of the genitalia were prepared using an Optec DV E3 630 digital camera attached to an Optec BK6000 microscope.

## ﻿Taxonomy

### ﻿Genus Thibetana

Taxon classificationAnimaliaLepidopteraZygaenidae

Efetov & Tarmann, 1995

BC81F2FC-2011-536D-90FA-6D5A32027B80

Thibetana Efetov & Tarmann, 1995: 74. Type-species: Artonasieversi Alphéraky, 1892, by original designation.

#### Diagnosis.

Fore- and hindwings with black ground colour and yellow markings, r2+r3 stalked. Uncus long and thin, valva fan-shaped, and saccus well-developed in male genitalia. Praebursa spherical, translucent, with ring-like sclerotization in female genitalia (Efetov and Tarmann 1995).

#### Distribution.

China, India.

##### ﻿Checklist of the genus of *Thibetana* Efetov & Tarmann, 1995

***Thibetanadelavayi* (Oberthür, 1894)**

*Artonadelavayi* Oberthür, 1894: 29; Alberti 1954: 280, pl. 29, fig. 9.

*Thibetanadelavayi*: Efetov and Tarmann 2017: 583; Efetov and Tarmann 2024: 431.

**Distribution.** China (Sichuan, Yunnan).

***Thibetanakeili* Efetov & Tarmann, 2017**

*Thibetanakeili* Efetov & Tarmann, 2017: 582, figs 1, 2, 7; Efetov and Tarmann 2024: 431.

**Distribution.** China (Xizang).

***Thibetanapostalba* (Elwes, 1890)**

*Artonapostalba* Elwes, 1890: 379, pl. 32, fig. 16.

*Thibetanapostalba*: Efetov and Tarmann 2017: 582, figs 3, 4, 8; Efetov and Tarmann 2024: 431.

**Distribution.** India (Sikkim).

***Thibetanasieversi* (Alphéraky, 1892)**

*Artonasieversi* Alphéraky, 1892: 5.

*Artonadejeani* Oberthür, 1894: 29.

*Artonagephyra* Hering, 1936: 1.

*Thibetanasieversi*: Efetov and Tarmann 1995: 74, figs 21, 22; Efetov 1997: 511, figs 4, 7, 10; Efetov and Tarmann 2024: 431.

**Distribution.** China (Qinghai, Sichuan).

***Thibetanaweii* Li & He, sp. nov.**

**Distribution.** China (Xizang).

***Thibetanawitti* Efetov, 1997**

*Thibetanawitti* Efetov, 1997: 509, figs 1, 2, 5, 6, 8, 9; Efetov and Tarmann 2024: 431.

**Distribution.** China (Xizang).

***Thibetanazebra* (Elwes, 1890)**

*Artonazebra* Elwes, 1890: 379, pl. 32, fig. 11.

*Thibetanazebra*: Efetov and Tarmann 2017: 583, figs 5, 6; Efetov and Tarmann 2024: 431.

**Distribution.** India (Sikkim).

##### ﻿Key to the genus of *Thibetana* Efetov & Tarmann, 1995

**Table:** 

1	Hindwing upperside with two yellow spots	**2**
–	Hindwing upperside with one yellow spot	**3**
2	Forewing upperside with one yellow spot at base	***T.weii* sp. nov.**
–	Forewing upperside with two yellow spots at base	***T.zebra***
3	Forewing upperside with a single or two yellow spots	**4**
–	Forewing upperside with four yellow spots	**5**
4	Forewing upperside with a single yellow spot	***T.delavayi***
–	Forewing upperside with two yellow spots	***T.sieversi***
5	Sacculus without process, juxta longer than uncus in male genitalia	***T.witti***
–	Sacculus with a round lobe, juxta shorter than uncus in male genitalia	**6**
6	Saccus as long as uncus in male genitalia	***T.keili***
–	Saccus twice as long as uncus in male genitalia	***T.postalba***

### ﻿ Thibetana weii

Taxon classificationAnimaliaLepidopteraZygaenidae

Li & He sp. nov.

6F4A5A5E-7409-5ED3-8E24-D19AB787EE4E

https://zoobank.org/B0F6360A-BF60-4222-93BD-D679E9DDB208

#### Type materials.

***Holotype*** • ♂, China, Xizang Autonomous Region, Mêdog County, the foot of Galongla Snow Mountain (29°44.2947'N, 95°40.6068'E), 3415 m, 31 July 2024, Weichun Li et al. leg. ***Paratype*** • 1 ♀, same data as holotype.

#### Diagnosis.

Forewing upperside with ovate yellow spot at base, two ovate spots near middle, and an 8-shaped yellow spot at distal part; hindwing upperside with subtriangular yellow spot and oblong yellow spot. In male genitalia, sacculus nearly rectangular, dentated on outer margin, ending with spine-like process on ventral margin.

#### Description.

**Figure 1.**
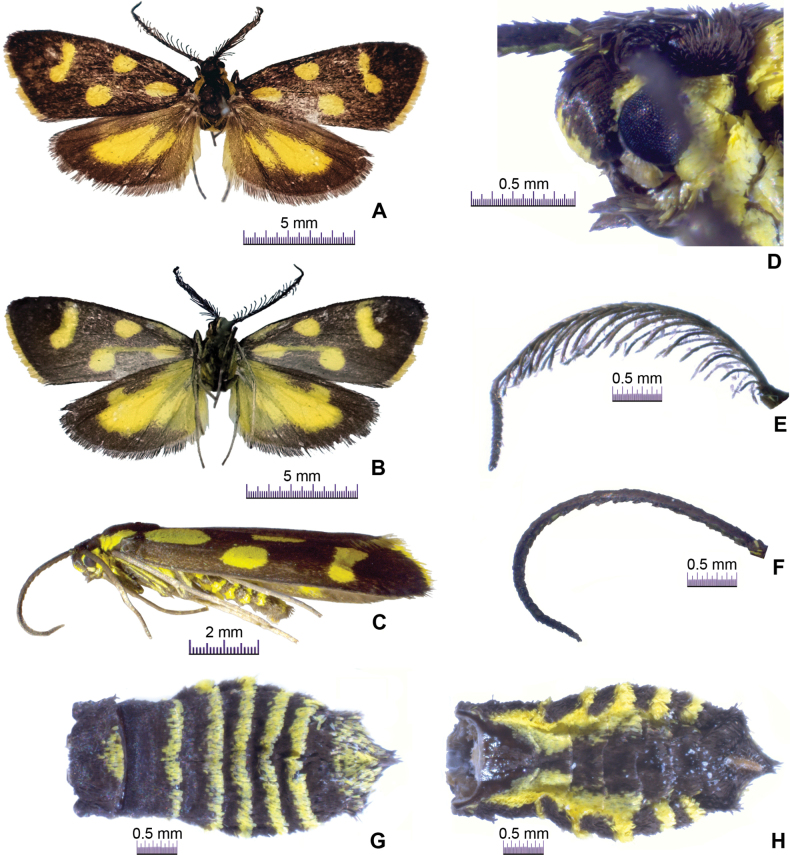
*Thibetanaweii* Li & He, sp. nov. **A** male habitus (holotype), dorsal view **B** ditto, ventral view **C** female habitus (paratype), lateral view **D** ditto, head, lateral view **E** male antenna (holotype), lateral view **F** female antenna (paratype), lateral view **G** female abdomen (paratype), dorsal view **H** ditto, ventral view.

***External morphology of imago*** (Fig. 1). Forewing length 8.5–9.0 mm. Frons yellow mixed black. Vertex black. Labial palpus approximately one and half as long as compound eye’s diameter, pale brown mixed with yellow. Male antenna pinnate except distal one-sixth serrate; female antenna serrate. Compound eye ovate, black, edged with yellow scales; ocellus round, black. Chaetosema well-developed, gray. Tegula yellow. Thorax black. Upperside of forewing black, bearing ovate yellow spot at base, two ovate yellow spots near middle, and an 8-shaped yellow spot at outer side of discoidal cell, cilia yellow; underside of forewing pattern same as upperside except for long and thin yellow stripe at basal one-fourth of costa, and longitudinal yellow stripe extending from basal one fourth to half part of forewing. Upperside of hindwing blackish-brown, with subtriangular yellow spot and oblong yellow spot, cilia blackish-brown; underside of hindwing yellow, costa, outer region, and apex blackish-brown. Legs greyish brown, femur yellow in lateral view. Dorsal side of abdomen blackish-brown, first segment covered with yellow scales in middle, second to sixth segments densely covered with yellow scales on distal margin, distal segment scattered with yellow scales; ventral side of abdomen blackish-brown, second to sixth segments densely covered with yellow scales near lateral margin.

**Figure 2.**
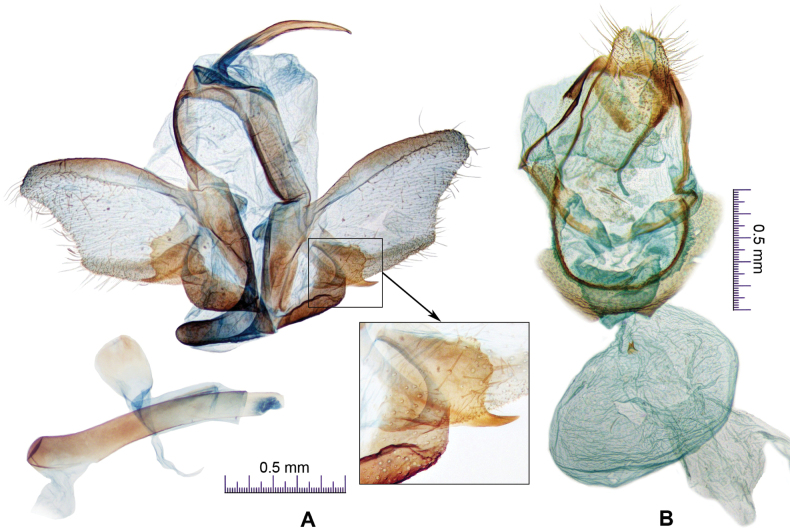
*Thibetanaweii* Li & He, sp. nov. **A** male genitalia (holotype), ventral view **B** female genitalia (paratype), ventral view.

***Male genitalia*** (Fig. 2A). Uncus thin and long, distal apex pointed. Tegumen arm slightly longer than uncus. Valva slightly broader near middle, distal one-third nearly triangular, and gently concave at approximately distal one-fourth on ventral margin; costa strongly sclerotized, reaching apex of valva; sacculus nearly rectangular, about one-fourth as long as valva, dentated on outer margin, ending with spine-like process on ventral margin. Saccus well-developed, as long as uncus, distal tip round. Juxta ovate. Phallus cylindrical as long as valva, without cornuti.

***Female genitalia*** (Fig. 2B). Papillae analis about two thirds as long as apophysis posterioris. Apophysis anterioris thin and long, nearly as long as apophysis posterioris. Praebursa spherical, translucent, with ring-like sclerotization. Ductus bursae inconspicuous. Corpus bursae ovate; signum small, bearing two spine-like projections; appendix bursae irregular shaped.

#### Etymology.

In honour of Dr. Fuwen Wei, a renowned conservation biologist, who contributes profoundly to biodiversity, zoological evolution and conservation biology. We suggest the Chinese common name as “魏氏藏斑蛾”.

#### Distribution.

China (Xizang).

#### Remarks.

The new species is similar to *Thibetanazebra* (Elwes, 1890) in the forewing pattern, but can be distinguished from the latter species by the upperside of the hindwing with a subtriangular yellow spot and an oblong yellow spot (Fig. 1A). In *T.zebra*, the second spot on the upperside of the hindwing is thin and long (Efetov and Tarmann 2017: fig. 5).

## Supplementary Material

XML Treatment for Thibetana

XML Treatment for Thibetana weii

## ﻿Additional information

### Author ORCIDs

Chao Jiang https://orcid.org/0000-0003-1841-1169

Weichun Li https://orcid.org/0000-0003-0154-861

### Data availability

All of the data that support the findings of this study are available in the main text.
